# “Comparing the effectiveness, acceptability and oral hygiene status between vacuum formed retainer and Begg’s retainer”: a pilot study

**DOI:** 10.1186/s12903-023-03010-1

**Published:** 2023-05-09

**Authors:** Payada Patnaik, Smruti Bhusan Nanda, Sumita Mishra

**Affiliations:** Institute of Dental Sciences, Siksha ‘O’ Anusandhan (Deemed to Be University), Kalinga Nagar, Bhubaneswar, 751003 Odisha India

**Keywords:** Acceptability, Begg’s retainers, Effectiveness, Oral hygiene, Retention, VFRs

## Abstract

**Background:**

Recently, Vacuum formed retainers (VFRs) are preferred as an Orthodontic retention appliance over conventional Begg’s retainers. Very few studies have been conducted between VFRs and Begg’s retainers. Hence, this study aims at assessing the effectiveness, oral hygiene and acceptability between VFRs and Begg’s retainers with a follow up period of 1 year.

**Methods:**

Eighty patients who completed fixed Orthodontic treatment were included. Retainers were delivered on the same day of debonding. Retainer 1/ R1 stands for VFRs and Retainer 2/ R2 stands for Begg’s retainers. The retainers were randomly allocated to both the arches. 40 VFRs and Begg’s retainers in maxillary and mandibular arch were given respectively.

Effectiveness, oral hygiene condition were performed at T_0_ (After debonding), T_1_ (3 months after using retainers), T_2_ (6 months after using retainers), T_3_ (9 months after using retainers), T_4_ (12 months after using retainers) follow up stages, except the feedback form and the breakage of retainers that were filled at T_4_ stage.

**Results:**

Both R_1_ and R_2_ retainers showed improvement in teeth alignment in both the arches at follow up stages. Interproximal contacts in maxillary and mandibular arch with VFRs and Begg’s retainers improved to 77.5% and 82.5% respectively. Considering the marginal ridge, Begg’s retainers and VFRs showed 95%, 55% increased proportion at T_4_ respectively (*p* < 0.05). Patients wearing Beggs’s retainers had significantly better (*p* < 0.05) oral hygiene status.

Significant differences were observed with Begg’s retainers in teeth biting, whereas no significant difference was found with fitting of appliance (*p* = 0.180) and gingival irritation (*p* = 1.000). VFRs were well accepted aesthetically that was significant. Retainers were prone to breakage but was not significant (*p* = 0.162).

**Conclusion:**

Begg’s wrap around retainers maintain good oral hygiene, improve the teeth alignment, interproximal contact and marginal ridges post Orthodontic treatment with better fitting of the appliance. VFRs are also preferred as they are good in maintaining proper teeth alignment with progressive improvement in the interproximal contacts and are aesthetically pleasing.

## Introduction

There are various goals of Orthodontic treatment. They can be expressed as achieving esthetics, stability, functional occlusion and aligned tooth. Active phase of Orthodontic movement takes one half to two years. Post the active phase, there are various changes noted in the occlusion. The negative changes are relapse and the positive changes are improved teeth interdigitation. Maintaining proper interdigitation of teeth is the most challenging stage of Orthodontic treatment. Avoiding relapse poses challenge to the Orthodontist, so thorough understanding of the factors associated with relapse is of paramount importance. In the 1960s, supra alveolar fibres were transected to prevent relapse. Orthodontically derotated teeth are more unstable. There are chances of rotational relapse even after a longer retention period. Other studies have shown that Circumferential Supracrestal Fiberotomy (CSF) could reduce dental relapse, especially of rotated teeth.

Hence, it is required for the clinician to have knowledge about the various methods of reducing relapse, advantages and disadvantages of various retainers. Retention appliances are fabricated to maintain teeth alignment and arch dimensions post Orthodontic treatment. Retainer is chosen by considering various factors like efficiency, cost, patient preferences, cooperation and satisfaction [1].

The prime concern and debate in the branch of Orthodontics is related to long term establishment of the achieved tooth movement. Commonly used retainers are the Hawley’s retainers (HRs), wrap around or Begg’s retainers, lingual bonded retainers and the newly familiarized Vacuum formed retainers (VFRs). The Hawley retainer was designed by Charles Hawley and is most popular as a removable retention appliance. The VFR was designed in 1971. Recently, VFRs are preferred as an Orthodontic retention appliance over conventional Begg’s retainers as they claimed to be aesthetic, durable, reduced failure rate, translucent [1, 2], inexpensive [3], comfortable [4] and simple to fabricate [5].

There are several studies relating the comparison between VFRs and HRs [6, 7]. Although retention is a must factor for successful orthodontic treatment, there is less evidence regarding the most appropriate strategy. Some have conducted randomized clinical trials showing VFRs to be more effective than HRs where as other studies have shown no statistical difference in the effectiveness of both the retainers. However, very few studies have been done regarding the effectiveness, acceptability, and oral hygiene between VFRs and Begg’s retainers.

### Aims and objectives

The aim of the present study was to compare the clinical effectiveness, oral hygiene status and acceptability of vacuum formed retainers (VFRs) and Begg’s retainers over a 12 months period post debonding, as very few cases come under less than 12 months retention period regimen. This was an experimental study with random allocation between two types of retainer. The hypothesis was that there was no comparison between the clinical effectiveness, oral hygiene status and acceptability of vacuum formed retainers ( VFRs) and Begg’s retainers over a 12 months period post debonding.

## Materials and methods

The study was approved by the Institutional Ethics Committee, Institute of Medical Sciences (IMS) and Sum Hospital, Siksha O Anusandhan University (Ref no./ DMR/IMSSH/SOA/180297).

Eighty patients who completed fixed Orthodontic treatment were included.

Inclusion criteria:

a Patients with fixed Orthodontic appliance with or without extraction
b Patients with optimum acceptable occlusion.
c Patients with good oral hygiene without any periodontal disease.
d Patients with age 18 years or above
e Patients with no prosthetic rehabilitation.

Exclusion criteria:

a Patients with missing teeth
b Patients who have undergone orthognathic surgery
c Patients with cleft lip and palate
d Patients with temporo-mandibular joint( TMD) disorders

**Fig. 1:**
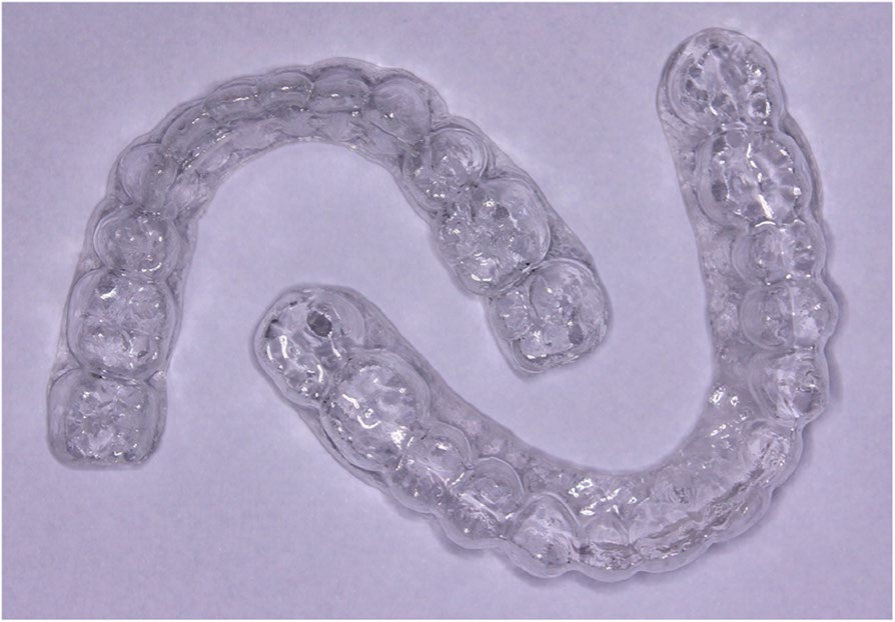
Essix retainer

**Fig. 2:**
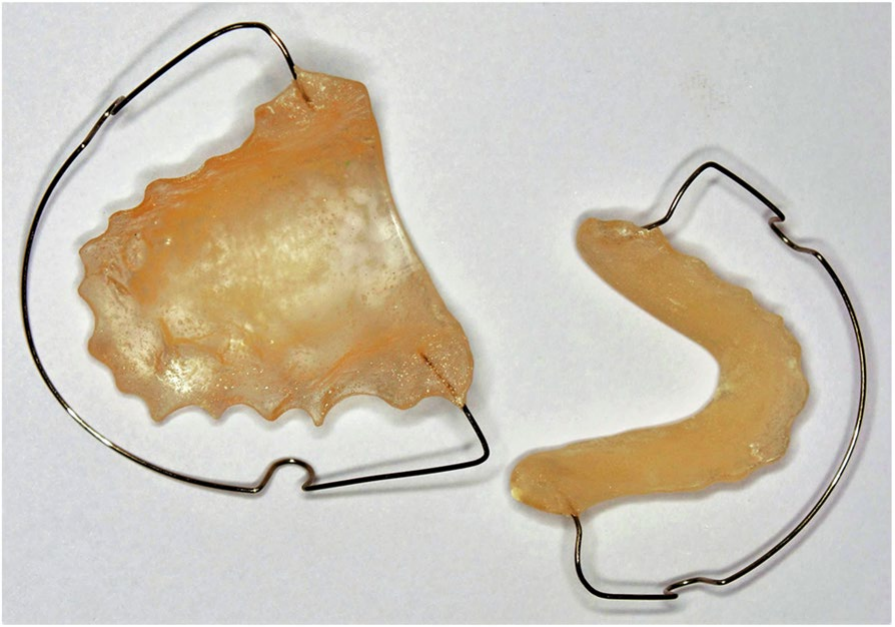
Begg’s retainer

**Fig. 3:**
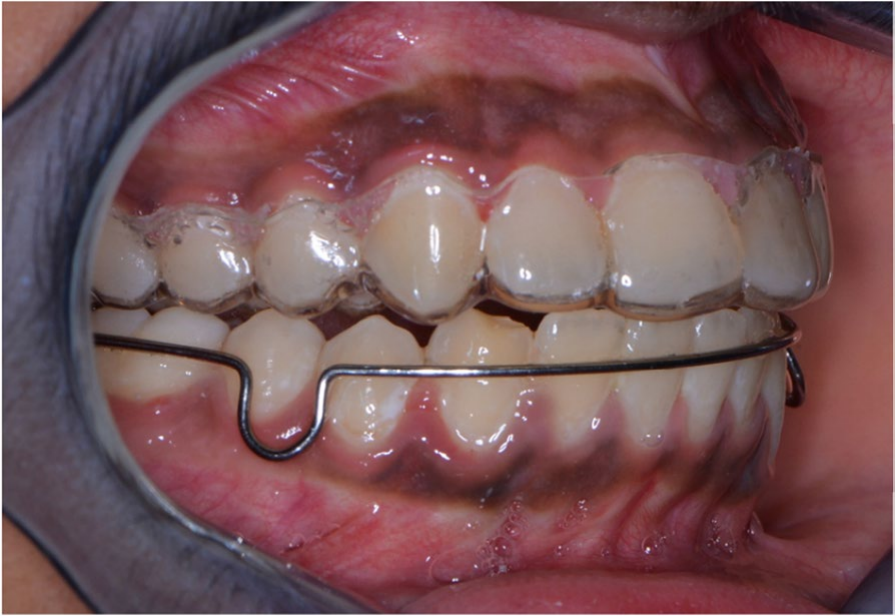
Retainers (buccal view)

After debonding of appliance and random allocation, the retainers were delivered on the same day. 40 VFRs, depicted as Retainer 1 / R1 (Fig. 1) were delivered in maxillary arch and mandibular arch. Similarly, 40 Begg’s retainers, depicted as Retainer 2 / R2 (Fig. 2) were delivered in maxillary and mandibular arch. The buccal view of the retainers is represented in Fig. 3. The patients were instructed to wear the appliance 24 h for the first 6 months followed by 6 months of night time wear. VFRs were fabricated with Duran 1.5 mm thickness sheet, composed of Polyethyleneterephthalate Glycol (PET-G) using Ministar machine (Scheu-Dental, Iserlohn, Germany). This material allows better withstanding wear without causing bite alterations. When the thermoformed plastic is thin, the premature occlusal contacts can be easily avoided (Sheridan et.al). Also, the Essix retainer has optimum fit, is esthetic and comfortable ensuring long-term stability of the occlusal alignment [8].

**Fig. 4:**
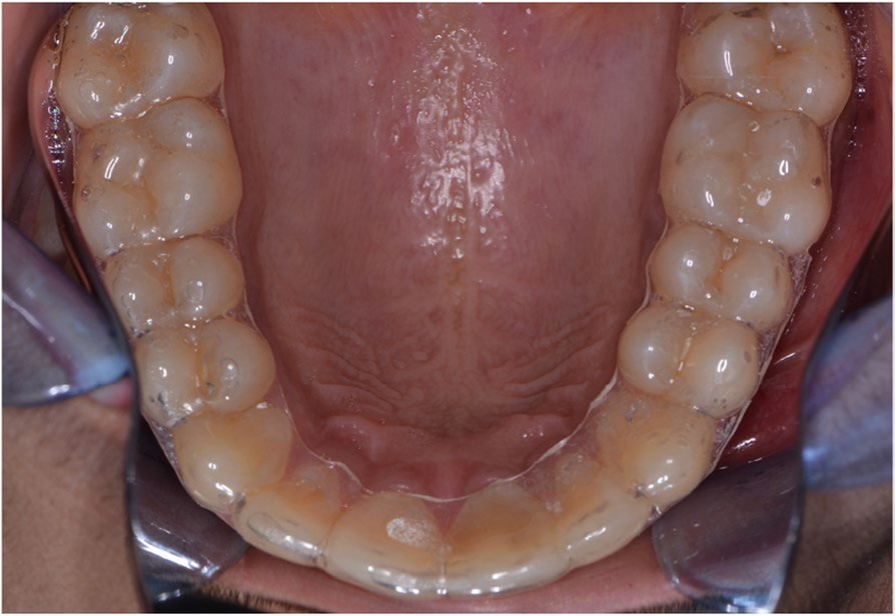
Occlusal coverage of VFR

The facial and buccal surfaces of VFRs were trimmed respectively to cover the incisal one-third of the incisors and occlusal surfaces of posteriors along with providing 2-mm buccal and 3-4 mm lingual extensions( Fig. 4). The retainers were extended till the last erupted molar [4, 9]. The Begg’s retainers were fabricated using acrylic baseplates ( DPI, heat cure acrylic, India) and labial bow with 0.9 mm stainless steel wire (Leone spa, Florence, Italy).

The labial bow has U loops placed between 1^st^ and 2^nd^ premolar in non-extraction cases, and between remaining premolar and 1^st^ molar in extraction cases which crossed the occlusal plane distal to the last erupted molar.

**Fig. 5:**
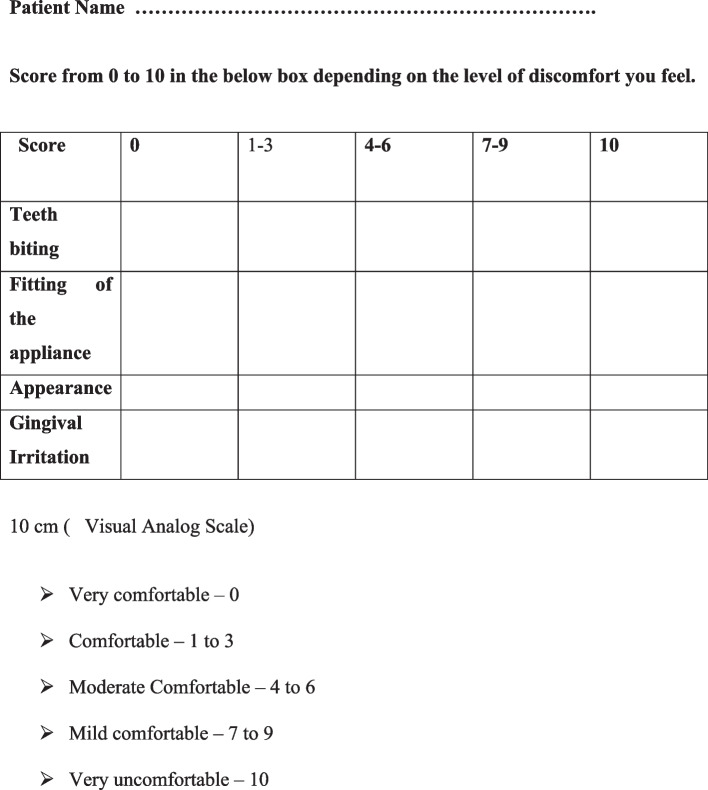
Questionnaire

Effectiveness of the retainers were measured using ABO measuring gauge. Oral Hygiene Index-Simplified index (OHI-S) and Gingival Index (GI) evaluated the patient’s oral hygiene condition, and patient’s compliance was evaluated by specific questionnaire (Fig. 5). All these findings were performed at T_0_ (After debonding), T_1_ (3 months after using retainers), T_2_ (6 months after using retainers), T_3_ (9 months after using retainers), T_4_ (12 months after using retainers) follow up stages, except the feedback form and the breakage of retainers that was filled at T_4_ stage.

Patients acceptance was evaluated using a 10-cm visual analogue scale (VAS) from the data collected in questionnaires that include 4 questions i.e. teeth biting (closing teeth with retainers, not chewing food), fitting of the appliance, appearance, gingival irritation. Patients were given instructions and explanations on how to complete these questionnaires. The lowest (least favourable) score was ‘0’ and the highest (most favourable) score was ‘10’. For example, if the retainer was very comfortable, it was scored as ‘0’. Questionnaires were filled in front of the Orthodontist at T4 stage.

### Sample size

The study envisages test of association of different characteristics (effectiveness, oral hygiene and acceptability) between retainers through chi-square test of independence. Therefore, minimum sample size is computed as per the requirement of the chi-square test of goodness of fit of contingency table.

**χ**^**2**^** tests—**Goodness-of-fit tests: Contingency tables

**Analysis:** A priori: Compute required sample size

**Input:** Effect size w = 0.41 (moderate effect size was assumed)α err prob = 0.05

Power (1-β err prob) = 0.80

Df = 5

**Output:** Noncentrality parameter λ = 12.9437000

Critical χ^2^ = 11.0704977

Total sample size = 77 Rounded off to 80.

Actual power = 0.8041224

Thus, the total sample size was 80.

For maxillary, mandibular arch minimum 40 R1 and R2 retainers from each were fabricated.

Thus, 160 retainers were designed.

Follow up:

Mean retention time was 1 year

Maxillary and mandibular casts were analysed at four stages:

- 1. T0 – Post debonding
- 2. T1 – After three months of using retainers
- 3. T2 – After six months of using retainers
- 4. T3—After nine months of using retainers

- 5. T4 – After twelve months of using retainers

Irrespective of the scheduled time, patient was asked to report to the department whenever there is breakage of the appliance. We ensured that all patients stuck to the recommended schedule of retainer wear, through periodic verbal reminders.

### Statistical analysis

Data collected on 80 cases were scrutinized, coded and entered into IBM SPSS 24.0 statistics, SPSS South Asia Pvt. Ltd. Data were analysed by the following statistical procedure.

1. Association of teeth biting, fitting of appliances, appearance, durability, gingival irritation, comfort of Maxillary and Mandibular arch in retainer 1 and retainer 2 were analysed using frequency procedure and Chi-square test / Fisher’s Exact test.
2. Association of oral hygiene with OHIS index and Gingival Index for maxillary and mandibular arch in retainer 1 and retainer 2 were analysed using frequency procedure and in retainer 1 and retainer 2 were analysed using frequency procedure and Chi / Fisher’s Exact test.
3. Comparison of Alignment, Marginal Ridges and Interproximal contact of effectiveness at T1, T2, T3, T4 with reference to T0 in maxillary arch and mandibular arch in retainer 1 and retainer 2 were analysed using frequency procedure and marginal homogeneity test.
4. The cut off value of ‘p’ for test of significance was taken as &it;0.05.

### Results

Both R_1_,R_2_ retainers showed improved teeth alignment in both the maxillary and mandibular arches (Table 1) at subsequent stages of follow up. Considering the marginal ridge changes (Table 1) with both retainers in maxillary arch; Begg’s retainers and VFRs showed 95%, 55% improved levelling at T_4_ respectively (*p* < 0.05). Improvement with VFRs at T_1_, T_2_, T_3_ when compared to T_0_ was not significant (*p* > 0.05) but it was significant for Begg’s retainers (*p* < 0.05). Marginal ridge changes with both retainers in mandibular arch; Begg’s and VFRs showed 87.5%, 52.5% improved levelling at T_4_ respectively (*p* < 0.05). At T_1_ with Begg’s retainers, improvement in marginal ridge when related to T_0_ was not significant (*p* = 0.157) but afterwards there was significant improvement over the time T_0_ (*p* < 0.05), but with VFRs at T_1_, T_2_, T_3_ the improvement with reference to T_0_ was not significant (*p* > 0.05). Interproximal contacts (Table 1) in maxillary arch with VFRs and Begg’s retainers reduced to 77.5% and 60% respectively (*p* < 0.05). It was also reduced to 75% at T_4_ with VFRs and 67.5% with Begg’s retainers in mandibular arch that was significant (*p* = 0.009) 7.

**Table 1:** Comparison on effectiveness of both retainers on alignment, marginal ridge and Inter-proximal contact over time for maxillary and mandibular arches

**A. Maxillary Arch**											
		T0		T1		T2		T3		T4	
		n (%)		n (%)	*p*-value	n (%)	*p*-value	n (%)	*p*-value	n (%)	*p*-value
Alignment	Retainer 1	23, 57.5	Reference Group	26, 65	0.02	28, 70	0.013	32, 80	0.001	34, 85	0.001
	Retainer 2	26, 65		30, 75	0.02	31, 77.5	0.011	33, 82.5	0.008	35, 87.5	0.003
Marginal Ridge	Retainer 1	17, 42.5		18, 45	0.157	20, 50	0.025	22, 55	0.011	22, 55	0.011
	Retainer 2	19, 47.5		24, 60	0.001	30, 75	< 0.001	31, 77.5	0.001	38, 95	< 0.001
Inter-proximal Contact	Retainer 1	30, 75		30, 75	1.000	33, 82.85	0.083	30, 75	0.617	31, 77.5	0.808
	Retainer 2	33, 82.5		34, 85	0.317	36, 90	0.083	28, 70	0.039	24, 60	0.006
**B. Mandibular Arch**											
Alignment	Retainer 1	26, 65	Reference Group	27, 67.5	0.083	30, 75	0.014	32, 80	0.005	31, 77.5	0.008
	Retainer 2	31, 77.5		31, 77.5	0.317	33, 82.85	0.046	33, 82.85	0.046	33, 82.85	0.046
Marginal Ridge	Retainer 1	18, 45		19, 47.5	0.317	19, 47.5	0.157	19, 47.5	0.083	21, 52.5	0.008
	Retainer 2	18, 45		19, 47.5	0.083	24, 60	0.001	25, 62.5	0.001	35, 87.5	< 0.001
Inter-proximal Contact	Retainer 1	36, 90		37, 92.5	0.317	38, 95	0.157	32, 80	0.102	30, 75	0.033
	Retainer 2	35, 87.5		35, 87.5	1.000	36, 90	0.317	32, 80	0.157	27, 67.5	0.009

Patients wearing Beggs’s retainers had significantly better (*p* < 0.05) OHI-S index and GI index in comparison to VFRs (Table 2). Significant differences (*p* = 0.000) were observed with Begg’s retainers in teeth biting,whereas no significant difference was found with fitting of appliance (*p* = 0.180) and gingival irritation (*p* = 1.000). For aesthetic appearance of patients, VFRs were well accepted, which was significant (*p* = 0.002). Results revealed that both the retainers were prone to breakage with subsequent follow ups but it was not significant (*p* = 0.162). Overall level of comfort was good with Begg’s retainers, but was not significant (*p* = 0.051) (Table3).

**Table 2:** Fisher’s exact test: association between the two retainers on hygiene maintenance through OHIS and Gingival Index

	Maxilla					Mandible				
	T0 (*p*-value)	T1 (*p*-value)	T2 (*p*-value)	T3 (*p*-value)	T4 (*p*-value)	T0 (*p*-value)	T1 (*p*-value)	T2 (*p*-value)	T3 (*p*-value)	T4 (*p*-value)
OHIS		0.102	< 0.001	< 0.001	< 0.001		0.136	0.049	0.018	< 0.001
Gingival Index	1	1	0.084	0.076	0.554	0.13	0.267	0.355	0.019	0.022

**Table 3:** Fisher’s exact test: comparison of the parameters (fitting, teeth biting, gingival irritation, appearance and breakage) between the retainers evaluated by the patients

	Fitting of the appliance (*p*-value)	Teeth biting with retainers (*p*-value)	Gingival Irritation (*p*-value)	Appearance of the retainers (*p*-value)	Breakage of retainers (*p*-value)
Maxilla	0.108	< 0.001	1	0.002	0.189
Mandible	0.108	< 0.001	1	0.002	0.348

## Discussion

Long term studies have shown that relapse occurs in approximately 70% of patients [1, 10]. To avoid relapse, the respiratory, masticatory and postural functional context is very important to correct if necessary. During the process of Orthodontic treatment plan, setting the retention protocol is of utmost importance [11]. Retention plan completes the comprehensive Orthodontic treatment [12, 13]. It should be the primary focus of Orthodontist to decide the best possible retention plan and retention device for individual patients.

There are variety of retention appliances available in Orthodontic literature. The operator should be extra cautious to decide on the retention appliance depending on the patients need and literature evidence.

Previous literature suggests Hawley’s [3, 9, 14, 15], VFRs [14, 16], Begg’s [17, 18] retainers as most efficient, effective and popular among Orthodontist. In our knowledge, this is the first ever study on the comparative assessment of compliance between two popularly used retainers over a period of one year post debonding.

HRs allows more vertical settling of posterior teeth than VFRs according to previous literature [19, 20]. It has a demerit of wire component crossing occlusally, which has issue especially in extraction cases. The side effect of spaces opening up interdentally is evident [21]. On this basis, Begg’s wrap around retainers have some advantage over the Hawley’s retainers, as there is absence of occlusally crossing wire components and uncovered occlusal surfaces that allow proper interdigitation of teeth. The biggest difference between VFRs and Begg’s retainers was found to be the presence of occlusal covering in VFRs, which leads to inadequate vertical settling of teeth during retention period [17, 22]. This aspect is beneficial in maintaining the levels of teeth that were moved in vertical direction to correct deep bite.

The literature evidence comparing VFRs and Begg’s wrap around is limited and the studies done earlier, were conducted for only a 6 month retention span [17]. The recommendation of retention period less than 12 months is very rare. Hence, this study was intended to do a thorough comparison between VFRs and Begg’s retainers focusing all important clinical aspects, over a period of 12 months. After the completion of Orthodontic treatment, debonding was done and R_1_, R_2_ retainers were delivered immediately. Patients were asked to wear the retainers for at least 12 months.

They were given a questionnaire to fill based on various aspect of comfort at the end of 12 months (T_4_). Three criteria from ABO model grading system to observe the retention of teeth were adopted to evaluate the retention effectiveness of both the retainers at T_0_, T_1_, T_2_, T_3_, T_4_. Also, the durability of the retainers was assessed by considering the breakage of the appliances reported by the patients. OHI-S index and GI index were recorded to evaluate the oral hygiene of the patients associated with R_1_ and R_2_ retainer at T_0_, T_1_, T_2_, T_3_ and T_4_.

### Association of effectiveness with R1 and R2 retainers

When considering alignment and contact point, both show improvement over the entire period of observation (Table 1), but the leveling of arches with proper marginal ridge alignment, is better with R_2_ retainer (Table 1). Whereas, in case of VFRs it is not improving over the observation period of 12 months. This is in agreement with previous literature by Sauget et al. (1997) [19] showing minimal improvement of teeth by R_1_ retainers in vertical direction during retention phase. Recent studies by author like Dincer and Isik Aslan (2010) [22], Hoybjerg et al. (2013) [20] too showed minimal improvement in vertical direction. The explanation of this is presence of thorough adapted occlusal covering of thermoplastic sheets in VFRs.

So, from the above finding it is clear that, decision of giving VFR retainers in finished Orthodontic treatment where the operator is expecting posterior settling post debonding, is contraindicated. VFRs are acceptable in cases where proper vertical positioning of teeth have been achieved before debonding. Proper posterior settling of teeth will deliver good posterior occlusal guidance resulting in distribution of occlusal forces on maximum number of inclined planes during oral functions, providing maximum periodontal support [23, 24].

When correlating *durability* between the two retainers, material thickness must be considered. Different authors have used various thickness of VFR sheets that may be of 0.75 mm, 1 mm and 1.5 mm. It was evident by Gardner et al [25] that VFRs material are more prone to wear and tear than retainers made with acrylic. Also, Hichens et al., [26] concluded increased number of Hawley’s retainers breakage than VFRs which could be due to thin acrylic plate. This might also be because of difference of elasticity between acrylic and thermoplastic materials used in the above discussed retainers.

After 12 months of retention plan, *durability* of the retainers was investigated. The results indicated breakage in both the retainers which was not statistically significant (Table 3). This finding is supported by the study of Sun et al [24]. However, in our study an increased breakage in R_1_retainers was found which could be due to stresses generated by forces exerted on the covered occlusal surfaces during its continuous wear [14]. The breakage of the R_2_ retainers was more commonly due to the mishandling and negligence by the patients.

### Association of oral hygiene with R_1_ and R_2_ retainers

At T_0_, immediately after debonding of fixed Orthodontic appliances, the patients had mild to moderate gingivitis based on Gingival Index (Table 2). In the presence of long standing fixed Orthodontic appliance, oral cavity is prone to accumulation of plaque and calculus leading to gingivitis [27]. It was found to be common in both the arches, but in the mandibular arch the severity was more as compared to maxillary arch. This was obvious because of the opening of mandibular salivary glands that makes mandibular anterior region more prone to accumulation of calculus [5]. Hence higher degree of gingivitis.

At T_1_, in maxillary and mandibular arch R_1_, R_2_ retainers deteriorated the gingival health, but was not statistically significant. However, it was more with R_1_ retainers (Table 2). Over time (towards the T_4_ stage), statistically significant worsening of the gingival health was observed in patients with R_1_ retainers. This is in accordance with the studies done by LiciaManzon et al., [28] that VFRs cover the entire teeth surface preventing salivary self-cleansing action intraorally, allowing growth of microorganisms resulting in poor oral hygiene. Moreover, minor inaccessible areas present in the appliance makes it cumbersome to clean, hence vulnerable to food lodgment, promoting microbial growth [29].

Analysis of simplified oral hygiene and gingival index statistically supports the concept of VFRs favoring debris and calculus formation, resulting in worsening of gingival conditions, compared with Begg’s retainers. Many factors influence the oral health status. Oral environment can be altered due to presence of these retention appliances which may change the micro flora. The menace is also related to the design, surface roughness of retainers and physical properties of materials [30].

### Association of comfort level with R_1_ and R_2_ retainers

The R_2_ retainers showed statistically significant acceptability in terms of *teeth biting* than R_1_ retainers (at T_4_) in both maxillary and mandibular arch (Table 3). It was apparent because in the design of R_2_retainers occlusal surfaces of the teeth are not covered, allowing better vertical settling of teeth over a period of time. Whereas, VFRs covering the incisal and occlusal surfaces retain the teeth in their debonded positions [5, 9].

When we evaluated *fitting of the appliance* with R_1_, R_2_ retainers over a period of 12 months; R_1_ retainers had statistically significant better fitting (Table 3). Reason being the VFRs are machine made, excluding human errors and accurate adaptation of VFR sheets over the intraoral hard and soft tissues. Thus, helping in a better fit of the appliance. The R_2_ retainers tend to loosen up over a period of time due to presence of malleable stainless steel wire components, which is also seen to be explained by *Kumar AG, Bansal A*in their study on Indian population comparing the effectiveness and acceptability between VRs and Begg’s retainer [17].

Results of statistical analysis was significant, suggesting R_1_ retainers being *aesthetically more acceptable* by patients than R_2_ retainers (Table 3). The rationale behind this is transparent plastic sheet and lighter weight appliance [19, 29]. However, Begg’s appliance was moderately accepted by patients and quite aesthetic as well.

Mild *Gingival irritation* was seen with R_1_and R_2_ retainers in both the arches which was statistically insignificant (Table 2). Lesser gingival irritation could be due to absence of retention clasp in both R_1_ and R_2_ retainers. This is also supported in a single-centre, randomized control trial by Mohammed Saleh et al. on-acceptability comparison between HRs and VFRs in Orthodontic adult patients [14].

The effect of resin based retainers on soft tissue must be understood. Studies have shown that uncured resins can leach and harm the soft tissues [31]. We have taken adequate precaution in terms of choice of material ( heat cured), employed proper mixing technique ( vacuum mixing) to minimize the presence of uncured monomers in the fabricated Begg’s retainer.

To conclude about the capability to retain the Orthodontic treatment outcome, a longer duration research is recommended. As the patients were given two different type of retainers in both maxillary and mandibular arch, the study was unable to provide insight regarding changes in occlusal contacts in vertical [23] and sagittal direction. As per previous studies by Mufide et al [22], and Wenjia et al [2], a newer study design having two groups of subjects with VFRs and Begg’s retainers in both the arches has to be observed to conclude the same. We have taken ABO measuring gauge, which is subjected to examiner inconsistency. It would have been much better to use a digital method [32, 33]. Also, a mouth breathing habit can provoke gingival inflammation of the anterior teeth but this breathing parameter was not taken into account.

"The outcome of our study recommends Begg’s wrap around retainers as a preferred mode of retention post Orthodontic treatment, as long as esthetics is not the prime concern.”

## Conclusions

Our study found that

1. Both VFRs and Begg’s retainers are efficient enough in maintaining proper teeth alignment but better leveling of marginal ridges is found with Begg’s retainers.
2. While considering only aesthetics, VFRs are largely preferred over Begg’s retainers.
3. VFRs deteriorate oral hygiene of the patients progressively over a period of time as compared to Begg’s retainers.
4. As long as durability of the retainers are considered, there is no statistically significant difference observed, but the number of breakages were more with VFRs.

The outcome of our study recommends Begg’s wrap around retainers as a preferred mode of retention post Orthodontic treatment, as long as esthetics is not the prime concern.

## Data Availability

All data generated or analysed during this study are included in this published article.
